# Biomarkers of Maternal and Fetal Exposure to Organochlorine Pesticides Measured in Pregnant Hispanic Women from Brownsville, Texas

**DOI:** 10.3390/ijerph10010237

**Published:** 2013-01-11

**Authors:** Ken Sexton, Jennifer J. Salinas, Thomas J. McDonald, Rose M. Z. Gowen, Rebecca P. Miller, Joseph B. McCormick, Susan P. Fisher-Hoch

**Affiliations:** 1 University of Texas School of Public Health, Brownville Regional Campus, 80 Fort Brown-AHC, Brownsville, TX 78520, USA; E-Mails: jennifer.j.salinas@uth.tmc.edu (J.J.S.); rmgowen@suclinica.org (R.M.Z.G.); joseph.b.mccormick@uth.tmc.edu (J.B.M.); susan.p.fisher-hoch@uth.tmc.edu (S.P.F.-H.); 2 School of Rural Public Health, Texas A&M System Health Science Center, SRPH Building, College Station, TX 77843, USA; E-Mail: tmcdonald@srph.tamhsc.edu; 3 Texas Commission on Environmental Quality, Region 12, 5425 Polk Street, Houston, TX 77023, USA; E-Mail: rebecca.miller@tceq.texas.gov

**Keywords:** biomarkers, fetal exposure, maternal exposure, organochlorine pesticides

## Abstract

Biomarkers of organochlorine pesticides were measured in both venous and umbilical cord blood from 35 pregnant Hispanic women living in Brownsville, Texas, USA. Gas chromatography with an electron capture detector was used to analyze specimens for 30 individual pesticides or their metabolites. Results indicate that blood concentrations were relatively low for most individual compounds, but that high-end (upper 10th percentile) values for total DDT were comparatively high. Although health effects associated with measured blood concentrations are uncertain, there is concern that fetal exposure to low levels of these OC compounds, either individually or in combination, might contribute to subsequent health problems, including neurodevelopmental effects, cancer, endocrine disruption, obesity and diabetes.

## 1. Introduction

Widespread use of organochlorine (OC) pesticides, such as dichlorodiphenyltrichloroethane (DDT), hexachlorobenzene (HCB), and hexachlorocyclohexane (HCH), has been drastically curtailed since the 1970s because of concerns about their environmental persistence, tendency to bioaccumulate and biomagnify, and possible adverse effects on humans and wildlife. The U.S. banned use of DDT (1972), HCH (1976), and HCB (1984), except for public health applications, and Mexico subsequently took similar action by banning HCB (1992) and phasing out both DDT and HCH (2000) [1,2,3,4]. Although levels in the environment have declined over time, concentrations of many OC pesticides or their metabolites (e.g., DDE—dichlorodiphenyldichloroethylene and DDD—(dichlorodiphenyldichloro-ethane), which are breakdown products of DDT) continue to be found in human tissue specimens from both U.S. [2,4,5,6,7,8] and Mexico [9,10,11].

Exposure to OC pesticides for U.S. residents occurs primarily from ingestion of contaminated food, including oily fish, shellfish, fatty meats, dairy products and fruits and vegetables. Contaminated drinking water (e.g., runoff from tainted soil) and air (e.g., long-range airborne transport) are comparatively minor exposure pathways [1,2,3]. Once inside the human body, OC pesticides and their metabolites tend to accumulate in adipose tissue, where they can remain for years. They also circulate in the lipid portion of blood serum [1,2,3,8]. Among the diverse toxic effects attributed to OC pesticides are carcinogenicity, male and female reproductive disorders, infertility, adverse developmental effects, neurotoxicity and immunotoxicity [1,2,3,12,13,14].

Research has documented that environmental chemicals in maternal blood can cross the placenta and produce fetal exposures [4,9,11,15,16,17,18,19]. The developing fetus and neonates are particularly prone to the adverse consequences of these exposures because they have higher rates of cell proliferation, decreased capability to activate and detoxify toxic compounds, and lower immune-response capacity [19,20,21]. Organochlorine pesticides or their metabolities have been reported in the blood of pregnant women [4,6,7,8,9,11] and in umbilical cord blood [9,11,15,16,17]. Research suggests that prenatal exposure to OC pesticides is associated with a variety of harmful outcomes [13], including delayed neurodevelopment [22], poor attention in early infancy [23], reduced birth size, weight, and head circumference [24,25], rapid weight gain in the first 6 months and elevated BMI later in infancy [26], obesity and type 2 diabetes later in life [27,28,29,30], and delayed age at menarche [31].

In this article, we summarize results of OC pesticide measurements in matched-pairs of maternal and cord blood from pregnant Hispanic women residing in Brownsville, Texas. Participants in the study were volunteers recruited from the patient population at a private clinic in Brownsville, which is located in south Texas along the U.S.-Mexico border, in a region known as the Lower Rio Grande Valley (LRGV). According to the U.S. Census Bureau [32], the city has a population of 172,434, of whom 92% are Hispanic. The 2006 American Community Service Survey ranked Brownsville as the most impoverished city in the nation based on average-annual household income [32]. More than a third of residents are 18 years old or younger, and 45% live in poverty; the highest proportion of any city in the U.S. with a population over 100,000 [32].

## 2. Methods

### 2.1. Subjects

Pregnant women in their first or second trimester presenting at a private gynecological clinic were told about the study and invited to participate. Informed verbal and written consent (either in Spanish or English, as appropriate) was obtained from those who agreed. No incentives were provided to participants, and the study received approval from the Committee for the Protection of Human Subjects at the University of Texas Health Science Center at Houston. Participants completed a short questionnaire on demographic and socioeconomic characteristics at the time of enrollment.

### 2.2. Specimen Collection and Handling

Between October 2005 and February 2006, venous blood samples were collected during routine clinic visits (third trimester) and cord blood was obtained at birth. The time between collection of maternal blood and collection of cord blood varied, with six matched maternal-cord sample pairs obtained within 24 h of each other, 10 within 2–14 days, 16 within 15–35 days, and three within 43–57 days. Collection of maternal blood was accomplished by venipuncture, and samples were put into a 10 mL, red-topped, vacutainer tube, then labeled, and refrigerated. After the umbilical cord was severed at birth, approximately 10 mL of blood were drained into a red-topped plain vacutainer tube, which was capped, labeled, and refrigerated. For shipping, each unopened blood tube was sealed with Teflon tape and placed upright in an individual slot inside a pressure jar. Gel or ice packs were placed under, around, and over the jar, which was then sealed in a shipping container and shipped by overnight express to the laboratory.

### 2.3. Laboratory Analysis

All samples were analyzed for 30 OC pesticides/metabolites in the laboratory at the School of Rural Public Health, Texas A&M University, in College Station, Texas [33]. The term “OC analyte” is used in the subsequent discussion to describe both the pesticides and their metabolites. Analyses were performed on whole blood and samples were not centrifuged prior to extraction. Dichloromethane was added to the blood samples to start the extraction process once they arrived at the lab. Later, sodium sulfate was added and the mixture homogenized three times at 3 min per extraction. The combined extracts were filtered and concentrated at 3.0 mL. The samples were then cleaned on a silica/alumina column and subjected to high-performance liquid chromatography (HPLC). Afterward, samples were concentrated to the final volume of 0.5 mL. All blood samples were subsequently analyzed for 30 individual OC pesticides or their metabolites using gas chromatography with an electron capture detector (GC/ECD) according to modified U.S. Environmental Protection Agency SW-846 8081A [34].

### 2.4. Limits of Detection

The limit of detection (LOD) for OC analytes varied from 0.50 to <1.00 ng/mL (three compounds—aldrin, 4,4’-DDD, endosulfan II), ≥1.00 to ≤1.75 ng/mL (26 compounds), and one compound (1,2,4,5-tetrachlorbenzene) had an LOD of 2.72 ng/mL (See Appendix). A significant proportion of blood samples often fall below the LOD in exposure biomarker studies, which necessitates application of simple yet valid methods for reporting and statistically analyzing concentrations <LOD [2,35]. All OC analyte concentrations measured as part of this study fell into one of three categories: >LOD; >0 but <LOD; and no instrument response (*i.e.*, no analyte detected). Statistical analyses used the instrument-generated values for the first two categories, and one-half of the lowest measurable concentration (0.005 ng/mL) for the third category.

### 2.5. Blood Concentrations Expressed on a Wet-Weight Basis (per Unit Volume of Serum)

Historically, varying laboratory practices have been used to express OC pesticide concentrations in blood [36,37]. Most commonly, investigators report measurements on either a wet-weight basis (per unit of serum volume) or as lipid-adjusted values (OC serum concentration ÷ serum lipids). But lack of scientific knowledge about the underlying biological interrelationships among adipose tissue concentrations, serum OC levels, and serum lipid values makes it difficult, if not impossible, to ascertain which method is most appropriate for the situation at hand. It has been shown, for example, that lipid-adjusted concentrations can be “highly prone to bias” when used in statistical causal models relating tissue concentrations to adverse health outcomes [37]. For the 35 matched-pairs of nonfasting blood specimens collected in this study, all blood concentrations of OC analytes are expressed on a wet-weight basis as quantity per unit volume of serum (ng/mL).

## 3. Results

A summary of demographic and socioeconomic characteristics for the 35 women enrolled in the study is presented in Table 1. All the women were Hispanic, between the ages of 18 and 38 years old, and had lived in the Brownsville area for several years. Only one woman identified herself as an active smoker. The mean height was 5.2 ft and mean weight was 161 lbs. The women had an average of 2.6 children at home and the average number of previous pregnancies was 2.8. Approximately 63% were born in the U.S., 6% in Mexico, and 31% elsewhere or unknown. Twenty percent graduated from high school or completed a GED, and an additional 49% graduated from a college or university. Seventy-one percent were married, 26% had never been married, and 3% were separated. Forty percent worked in an office, business, or shopping mall, 20% were housewives, and 20% were teachers, administrators, or students.

Cord and maternal blood were analyzed for 30 OC analytes, including four isomers (α, β, γ, and ∆) of hexachlorocyclohexane (HCH) and several compounds that are components of commercial DDT (dichlorodiphenyltrichloroethane) and/or metabolites of DDT (*i.e.*, 2,4’-DDT and 4,4’-DDT, 2,4’-DDE and 4,4’-DDE (1,1’-(2,2-dichloroethenylidene)-*bis*[4-chlorobenzene]), 2,4’-DDD and 4,4’-DDD (1,1’-(2,2-dichloroethylidene)-*bis*[4-chlorobenzene])). As shown in Table 2, 14 compounds (47%) were not found at all and 11 compounds (37%) were not measurable in more than 75% of the specimens. These 25 compounds were excluded from further statistical analysis, but are included (if applicable) as part of the “total HCH, total chlordane, and total DDT” values (sum of all measureable concentrations above zero) reported in Table 3.

**Table 1:** Sociodemographic attributes of women participating in the study (N = 35).

VARIABLE	MEAN (S.D., Range)
Age (years)	25.8 (5.5, 18–38)
Height (feet)	5.2 (0.21, 4.8–5.6)
Weight (pounds)	160.9 (36.9, 96–237)
Previous Pregnancies	2.8 (0.76, 2–4)
Number of Children	2.6 (0.5, 2–3)
**VARIABLE**	**NUMBER OF WOMEN (%)**
**Country of Birth**	
United States	22 (62.9)
Mexico	2 (5.7)
Other or Unknown	11 (31.4)
**Education**	
Middle School	2 (5.7)
Some High School	8 (22.9)
Graduated High School/GED	7 (20.0)
Graduated College/University	17 (48.6)
Unknown	1 (2.9)
**Occupation**	
Housewife	7 (20.0)
Office/Business/Shopping Mall	14 (40.0)
Teacher/Student/Administrator	7 (20.0)
Outdoor Job	1 (2.9)
Unemployed	1 (2.9)
Other	4 (11.4)
Unknown	1 (2.9)
**Marital Status**	
Married	25 (71.4)
Never Married	9 (25.7)
Separated	1 (2.9)

**Table 2:** Summary of OC analytes either not found or infrequently measurable in maternal and cord blood.

No Concentration Measureable in 100% of Blood Samples			
Aldrin	2,4’-DDE		Endsulfan I
Endrin	1,2,3,4-Tetrachlorobenzene		Endosulfan II
Alpha-Chlordane	1,2,4,5-Tetrachlorobenzene		Endosulfan Sulfate
Gamma-Chlordane	Pentachloroanisole		Chlorpyrifos *
Alpha-HCH	Pentachlorobenzene		
**No Concentration Measureable in >75% to ≤99% of Blood Samples**			
Dieldrin		Delta-HCH	
Heptachlor		Gamma-HCH	
Oxychlordane		2,4’-DDD	
Cis-Nonachlor		4,4’-DDD	
Beta-HCH		2,4’-DDT	
		Mirex	

* organophosphate pesticide.

**Table 3:** Summary statistics for OC concentrations (ng/mL) in matched pairs of maternal and cord blood (N = 35).

COMPOUND	CORD BLOOD		MATERNAL BLOOD		CORD/MATERNAL RATIO	
	GM (GSD)	AM (SD)	GM (GSD)	AM (SD)	GM (GSD)	AM (SD)
Heptachlor-Epoxide	0.03 (1.03)	0.06 (0.07)	0.03 (1.03)	0.05 (0.06)	1.07 (1.16)	1.08 (0.18)
*trans*-Nonachlor	0.01 (1.01)	0.01 (0.01)	0.02 (1.02)	0.04 (0.09)	0.54 (4.62)	1.30 (2.49)
4,4’-DDE	0.22 (1.25)	0.30 (0.30)	0.82 (2.27)	1.23 (1.26)	0.27 (1.88)	0.33 (0.23)
4,4’-DDT	0.01 (1.01)	0.01 (0.01)	0.01 (1.01)	0.02 (0.01)	0.82 (2.25)	1.09 (0.97)
HCH	0.02 (1.02)	0.02 (0.03)	0.02 (1.02)	0.03 (0.04)	0.79 (1.84)	0.89 (0.31)
Total HCH	0.02 (1.02)	0.03 (0.05)	0.02 (1.02)	0.03 (0.04)	1.07 (5.32)	4.07 (8.24)
Total Chlordane	0.03 (1.02)	0.07 (0.08)	0.04 (1.04)	0.10 (0.14)	0.65 (3.08)	0.89 (0.46)
Total DDT	0.24 (1.27)	0.31 (0.30)	0.83 (2.29)	1.25 (1.29)	0.29 (1.93)	0.35 (0.25)

GM = geometric mean; GSD = geometric standard deviation; AM = artithmetric mean; SD = standard deveiation; DDE = dichlorodiphenyldichloroethylene; DDT = dichlorodiphenyltrichloroethane; HCH = hexachlorocyclohexane; Total HCH = sum of all HCH isomer concentrations > 0 (including those not reported in the table); Total Chlordane = sum of all Chlordane isomer concentrations > 0 (including those not reported in the table); Total DDT = sum of all DDT-related compounds with concentrations > 0 (including those not reported in the table).s

Summary statistics for the other five compounds (heptachlor-epoxide, *trans*-nonachlor, 4,4’-DDE, 4,4’-DDT, and hexachlorobenzene) and for total HCH, chlordane, and DDT (sum of all concentrations above zero for relevant isomers and/or metabolites of each compound) are provided in Table 3. Mean and standard deviation for each compound are presented for cord blood, maternal blood, and the ratio of cord-to-maternal blood (C/M ratio). The geometric mean C/M ratio was <1 for trans-nonachlor, 4,4’-DDE, 4,4’-DDT, hexachlorobenzene, total chlordane and total DDT, and slightly >1 for heptachlor-epoxide and total HCH. Based on calculated z-scores, differences between matched cord and maternal blood were statistically significant (*p* < 0.05) only for 4,4’-DDE and total DDT.

Although concentrations of individual analytes were comparatively low overall, total maternal DDT levels were comparatively higher for those in the upper tail of the distribution. For the four highest maternal specimens (approximately the upper tenth percentile), total DDT concentrations ranged from 4.29–4.74 ng/mL, while the four lowest values were less than 0.30 ng/mL (a spread of more than 14X). Total HCH and total chlordane concentrations were uniformly low, with maternal values ≤0.15 ng/mL for HCH and ≤0.55 ng/ml for chlordane.

## 4. Discussion and Conclusions

Results from this study, which involved women in higher-than-average socioeconomic strata, show that pregnant Hispanic women and their fetuses residing in Brownsville are differentially exposed to a diversity of OC pesticides, and concentrations in maternal blood are typically similar to or higher than in cord blood. Previous studies have used a variety of analytical techniques to monitor disparate populations, and relatively few measurements are available from Mexican-American women and their fetuses. It is not straightforward, therefore, to put measured OC concentrations into perspective by relating them to comparable statewide or national distributions. The Centers for Disease Control and Prevention’s (CDC’s) Fourth Report on Human Exposure to Environmental Chemicals [2] summarizes blood OC data from several hundred adult Mexican Americans (both male and female) collected between 1999 and 2004. Similar to the data from Brownsville, CDC’s geometric mean for heptachlor-epoxide, DDT, and HCH tended to be below the LOD, while for *trans*-nonachlor it was approximately 0.65 ng/mL (compared to 0.01 ng/mL in Brownsville) and for DDE it was about 3.5 ng/mL (compared to 0.22 ng/mL for 4,4’-DDE in Brownsville).

Although mean values for Brownsville appear to be relatively low, high-end concentrations (upper 10%) for total DDT are relatively high. There was considerable spread in the Brownsville data for total DDT—the range was from <0.15 ng/mL for both cord and maternal specimens to 1.47 ng/mL for cord blood and 4.74 ng/mL for maternal blood. The four highest total DDT values (roughly the top 10th percentile) measured in Brownsville varied from 0.54 to 1.47 ng/mL for cord blood and from 4.29 to 4.74 ng/mL for maternal blood. The maternal concentrations were approximately four times higher than 95th percentile values reported by the CDC [2], while cord blood concentrations were similar.

Based on current scientific knowledge and understanding, the human health effects from these blood levels of OC pesticides are unknown. Keeping in mind that our cohort is a relatively-small convenience sample, and that quantifiable OC levels were often below the nominal limit of detection, our findings, nevertheless, document both maternal and *in utero* exposure to a variety of OC pesticides, many of which are known or suspected neurodevelopmental toxicants, human carcinogens, endocrine disrupters, and obesogens [1,2,3,21,22,23,24,25,26,27,28,29,30]. This raises questions about the potential harmful effects of observed OC exposures on the developing fetus, especially given its inherent biological vulnerability, the potential for synergistic interactions between multiple chemical and nonchemical stressors, and the likelihood that susceptibility is enhanced by socioeconomically-difficult and environmentally-demanding living conditions [18,19,20,21,27,28,29,30,38,39,40,41,42].

More than 90% of Brownsville residents are Hispanic (primarily Mexican American), and many exhibit socioeconomic characteristics associated with poor health outcomes, including poverty, illiteracy, inadequate housing, unemployment, English as a second language, substandard diet, lack of access to health care and generally more stressful and less healthful lives [43,44,45]. Residents along the U.S.-Mexico border often lack adequate sewage treatment facilities, cannot afford air conditioning, experience frequent flooding, and do not have sufficient knowledge about healthy lifestyles [43,44]. Among environmental hazardous routinely encountered by this population are (a) contaminated drinking and recreational water from agricultural runoff, municipal waste, and factory discharges; (b) polluted air caused by emissions from motor vehicles, industrial facilities, agricultural operations, and open burning of trash; (c) tainted food related to subsistence farming and fishing, and (d) contaminated soil resulting from pesticide use, waste disposal, and illicit scrap yards and tire dumps [46]. Moreover, Brownsville has a higher-than-average prevalence of many chronic diseases to which environmental exposures contribute, including tuberculosis, cardiovascular disease, obesity and diabetes, [43,44,45], and children living along the Texas-Mexico border are hospitalized with asthma at a 36% higher rate than non-border children [47]. The evidence suggests that pregnant women (and their fetuses) living in Brownsville are likely to experience varying exposure to a complex mixture of chemical and nonchemical stressors [43,44,45,46,47,48]; a situation that could make them more susceptible to the adverse effects of OC pesticides and other environmental chemicals [38,39,40,41,42].

Because OC pesticides are toxic to humans, persist in the environment for years, bioaccumulate in the body and have a tendency to biomagnify in the food chain, their use has been banned or restricted in most developed countries for more than 30 years [1,2,3,4]. The 2001 Stockholm Convention, a global treaty signed by more than 150 countries, called on governments to eliminate the production and use of 13 OC pesticides (aldrin, chlordane, chlordecone, dieldrin, endrin, heptachlor, HCB, alpha- and beta-HCH, lindane, mirex, pentachlorobenzene, toxaphene) and reduce the manufacture and application of DDT [49]. Nevertheless, as a result of residues from past applications, continued use by some countries, and long-range environmental transport, OC pesticides and related by-products are still routinely detected in air, water, soil, sediment, fish, birds, and mammals from around the World [1].

Human biomonitoring studies have documented OC pesticides and their metabolites in blood from people worldwide [15,16,17,26], including those living in Mexico [9,10,11] and the U.S. [2,4,5,6,7,8]. Maternal exposures and related cross-placental transport are a particular public health concern because the developing fetus is intensely sensitive to xenobiotic chemicals during certain time windows of vulnerability when even minute amounts of exogenous substances can produce harmful consequences [7,13,18,19]. The data on hand indicate that fetal exposures to OC pesticides/metabolites occur regularly [9,10,11,15,16,17,19] and may be associated with neurodevelopmental problems [13,22,23], decreased birth size, weight and head circumference [24,25], quick weight gain and elevated BMI in infancy [26], and subsequent obesity, pre-diabetic conditions, and type 2 diabetes [27,28,29,30].

Future research needs to involve a larger, statistically-based sample, which includes pregnant women from lower socioeconomic strata, and more detailed information should be collected on participants’ diet, housing characteristics, living situation, neighborhood environment, and activity patterns. Follow-up efforts ought to be aimed at identifying exposure pathways and routes for those at the high-end of the exposure distribution. Because of the elevated prevalence of obesity and diabetes in this population, particular attention should be directed towards investigating the relationship between fetal exposure to chemical obesogens, such as DDT, and consequent development of childhood obesity and pre-diabetic conditions. The cumulative effect of fetal exposure to multiple chemical and nonchemical stressors needs to be examined in order to identify those individuals at highest comparative risk for adverse health outcomes.

## Appendix

**Table A1:** Limits of detection (LOD) for 30 organochlorine compounds examined in cord and maternal blood specimens.

Organochlorine Compound	Limit of Detection (LOD) ng/mL
Aldrin	0.89
Dieldrin	1.27
Endrin	1.52
Heptachlor	1.57
Heptachlor-Epoxide	1.32
Oxychlordane	1.52
Alpha-Chlordane	1.07
Gamma-Chlordane	1.11
*trans*-Nonachlor	1.16
*cis*-Nonachlor	1.09
Alpha-Hexachlorocyclohexane (α-HCH)	1.28
Beta-Hexachlorocyclohexane (β-HCH)	1.25
Delta-Hexachlorocyclohexane (∆-HCH)	1.38
Gamma-Hexachlorocyclohexane (γ-HCH)	1.24
2,4’-DDD	1.62
4,4’-DDD	0.96
2,4’-DDE	1.00
4,4’-DDE	1.11
2,4’-DDT	0.87
4,4’-DDT	1.24
1,2,3,4-Tetrachlorobenzene	1.04
1,2,4,5-Tetrachlorobenzene	2.72
Hexachlorobenzene	1.76
Pentachloroanisole	1.53
Pentachlorobenzene	1.43
Endosulfan II	1.73
Endosulfan I	1.73
Endosulfan Sulfate	0.49
Mirex	1.26
Chlorpyrifos (organophosphate pesticide)	0.54
